# Transcriptomic Analysis of *Mecp2* Mutant Mice Reveals Differentially Expressed Genes and Altered Mechanisms in Both Blood and Brain

**DOI:** 10.3389/fpsyt.2019.00278

**Published:** 2019-04-29

**Authors:** Albert Sanfeliu, Karsten Hokamp, Michael Gill, Daniela Tropea

**Affiliations:** ^1^Neuropsychiatric Genetics, Department of Psychiatry, School of Medicine, Trinity Translational Medicine Institute, St James Hospital, Dublin, Ireland; ^2^Department of Genetics, School of Genetics and Microbiology, Smurfit Institute of Genetics, Trinity College Dublin, Dublin, Ireland; ^3^Department of Psychiatry, School of Medicine, Trinity College Institute for Neuroscience, Trinity College Dublin, Dublin, Ireland

**Keywords:** Rett syndrome, methyl-CpG-binding protein 2, gene expression, transcriptomics, cerebellum, blood

## Abstract

Rett syndrome is a rare neuropsychiatric disorder with a wide symptomatology including impaired communication and movement, cardio-respiratory abnormalities, and seizures. The clinical presentation is typically associated to mutations in the gene coding for the methyl-CpG-binding protein 2 (*MECP2*), which is a transcription factor. The gene is ubiquitously present in all the cells of the organism with a peak of expression in neurons. For this reason, most of the studies in Rett models have been performed in brain. However, some of the symptoms of Rett are linked to the peripheral expression of *MECP2*, suggesting that the effects of the mutations affect gene expression levels in tissues other than the brain. We used RNA sequencing in *Mecp2* mutant mice and matched controls, to identify common genes and pathways differentially regulated across different tissues. We performed our study in brain and peripheral blood, and we identified differentially expressed genes (DEGs) and pathways in each tissue. Then, we compared the genes and mechanisms identified in each preparation. We found that some genes and molecular pathways that are differentially expressed in brain are also differentially expressed in blood of *Mecp2* mutant mice at a symptomatic—but not presymptomatic—stage. This is the case for the gene *Ube2v1*, linked to ubiquitination system, and *Serpin1*, involved in complement and coagulation cascades. Analysis of biological functions in the brain shows the enrichment of mechanisms correlated to circadian rhythms, while in the blood are enriched the mechanisms of response to stimulus—including immune response. Some mechanisms are enriched in both preparations, such as lipid metabolism and response to stress. These results suggest that analysis of peripheral blood can reveal ubiquitous altered molecular mechanisms of Rett and have applications in diagnosis and treatments’ assessments.

## Introduction

Rett syndrome (RTT) is a rare neurological disease, affecting approximately 1 in every 10,000 live female births. Approximately 95% of RTT cases present with mutations in the *MECP2* gene, which is located in the long arm of the X chromosome (1). Its genomic location explains why the majority of patients are females. Females can compensate for loss of *MECP2* function with an extra intact copy on the homologous X chromosome, but this is not the case for males. Consequently, males have a severe phenotype and represent less than 1% of RTT patients.

The symptoms manifest after a period of apparent normality, corresponding to the first 6–18 months of life. After this stage, patients present neurological features (microcephaly, seizure), motor disability (ataxia, loss of purposeful hand use, stereotyped hand movements, loss of the ability to walk, hypotonia), social impairment (loss of speech, unresponsiveness to social cues, lack of emotional expression), and autonomic complications (respiratory anomalies, cardiac dysfunction, constipation) (2). The symptoms and their severity can be variable from one patient to another. One of the reasons for this variability is thought to be skewed X-inactivation, as patients with an X-inactivation biased to the nonmutated copy of *MECP2* have shown little to no symptoms (3).

The strong association between *MECP2* mutations and the disease has prompted the generation of mutant mice, which present specific mutations in *Mecp2* or a lack of its expression (4–10). These mice show signs that resemble the symptoms in patients; hence, they are considered valuable models for shedding light on the molecular mechanisms underlying RTT (4, 5).

*MECP2* encodes for methyl-CpG-binding protein 2 (MeCP2), a chromatin binding protein (11) that is expressed ubiquitously in the body with major expression in the central nervous system (CNS). As MeCP2 was first postulated as a transcriptional repressor, several groups used the mouse models to study gene expression changes (12–14). These studies have revealed that MeCP2 can both upregulate and downregulate gene expression, and that gene expression changes are specific to different brain areas and cell types (12–14).

Although *MECP2* is highly expressed in the brain, it is also present in several other tissues/organs, and a recent mouse model showed that a small portion of symptoms are still present when *Mecp2* is exclusively expressed in the CNS but not in the rest of the body (15), supporting the possibility that molecular signatures of dysfunctions in RTT may be present in peripheral tissues, and they are possibly linked to changes in the brain.

In our study, we used RNA sequencing to compare the differential gene expression in brain and in blood in a mouse model of RTT. This analysis reveals associations between genes expressed in the two tissues and has important applications in the detection of peripheral biomarkers for Rett syndrome.

## Results

### MeCP2 Protein Expression Levels Are High in Mouse Cerebellum at 7 Weeks of Age

In the brain, the expression of *Mecp2* is dynamically modulated during development (16, 17). Additionally, *Mecp2* expression can differ between brain areas (15), as well as the genes that *Mecp2* regulates (13). For these reasons, we understand that to perform a transcriptomic analysis, it is necessary to use a specific brain area, and that the area should ideally have high levels of MeCP2 expression at the developmental stage in which the study is conducted.

**Figure 1:**
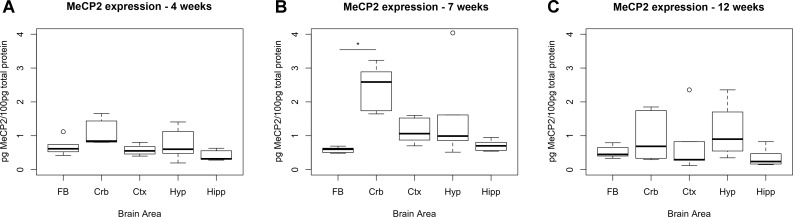
Expression levels of MeCP2 peak in cerebellum at 7 weeks. Expression of MeCP2 protein in full brain (FB), cerebellum (Crb), cortex (Ctx), hypothalamus (Hyp), and hippocampus (Hipp) of WT C57Bl/6 male mice, at the ages of 4 **(A)**, 7 **(B)**, and 12 weeks **(C)**. Six mice per group were examined. At 7 weeks, the cerebellum is the only area where MeCP2 protein expression is significantly different than the full brain. The data was analyzed with a Kruskal–Wallis test, considering significant p-values < 0.05. No significant differences between areas were found at 4 or 12 weeks of age.

To identify the ideal brain region for the developmental stage of our study (i.e., 7 weeks, when symptoms are advanced in the *Mecp2*-null male), we measured the temporal expression of MeCP2 protein in wild-type (WT) C57Bl/6 male mice in different brain areas (cortex, cerebellum, hippocampus, and hypothalamus) and compared them to the full brain expression (**Figure 1**). *P*-values were calculated using a Kruskal–Wallis test followed by a Dunn’s multiple comparisons test.

At 7 weeks, the only area that showed significantly higher levels of MeCP2 compared to full brain was the cerebellum (4.29-fold, *p*-value = 0.0002). We also screened for the expression of MeCP2 protein at 4 and 12 weeks of age, at which stage there were no significant differences between the full brain and the specific areas.

The results show that MeCP2 expression changes during development and at 7 weeks, the MeCP2 protein is highly expressed in the cerebellum.

### RNA Sequencing Reveals Differentially Expressed Genes in Cerebellum and Blood of *Mecp2-Null* Mice

**Figure 2:**
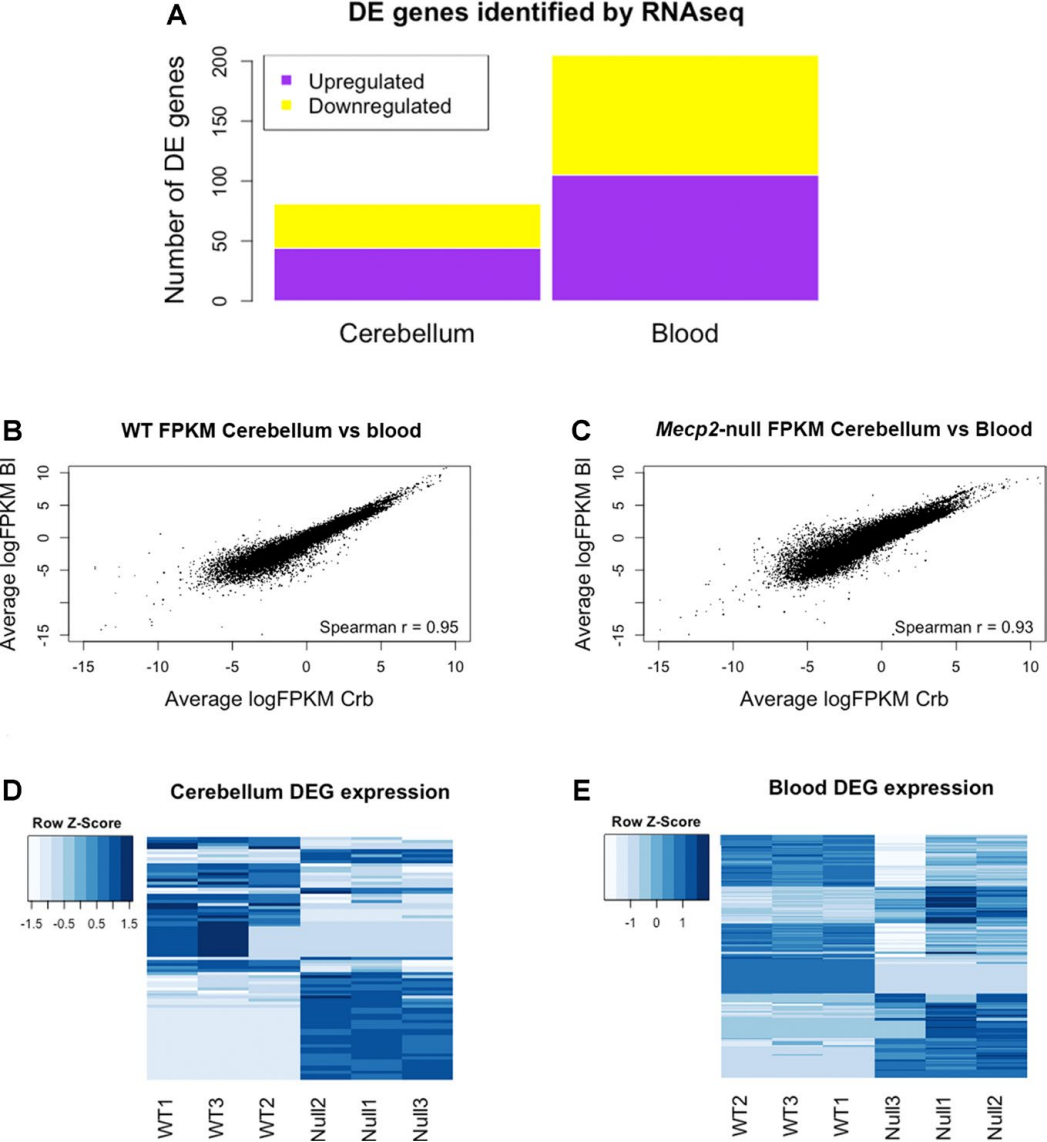
RNAseq analysis reveals differentially expressed genes (DEGs) in cerebellum and blood and identifies gene expression correlation between them. Summary of the *Mecp2*-null vs WT RNAseq analysis. **(A)** Number of DEGs identified in blood and cerebellum between *Mecp2*-null (N = 3) and WT mice (N = 3). 205 DEGs were identified in blood (105 upregulated and 100 downregulated), and 81 were identified in cerebellum (44 upregulated and 37 downregulated). For statistical comparison, we used EdgeR package with FDR correction and a significance level of p < 0.05. **(B, C)** Scatterplots of average gene expression in cerebellum versus average gene expression in blood, in logFPKM in WT **(B)** and *Mecp2*-null **(C)**. **(D, E)** Heat maps of expression Z-scores (normalized FPKM values) of the DEGs identified in cerebellum and blood.

RNA sequencing (RNAseq) was performed on male *Mecp2*-null mice at 7 weeks of age and compared to wild-type (WT) matched controls. The experiment was performed independently in cerebellum and blood. Tissue-dependent EdgeR analysis revealed 81 differentially expressed (DE) genes (DEGs) in cerebellum, of which 44 were upregulated and 37 downregulated (**Figure 2A**). In blood, 205 DEGs were found to be significantly different between WT and mutants: 105 upregulated and 100 downregulated. We found similar levels of gene expression in both tissues (R^2^ Spearman WT = 0.95, R^2^ Spearman MUT = 0.93, **Figure 2B and C**). DEGs show different profiles in the heatmaps of expression (**Figure 2D and E**). **Table 1** contains the first 10 genes of each group ordered by *p*-value. The full list is available in **Supplementary File 1**. As an intrinsic control, we checked the expression of *Mecp2*, and we confirmed a strong difference between WT and mutants: a log2 fold change (log2FC) of −3.88 in the cerebellum of *Mecp2*-null mice relative to the controls, with an FDR-corrected *p*-value [false discovery rate (FDR)] of 5.38E-27. In blood, *Mecp2* showed a log2FC of −2.50, and an FDR of 4.07E-06. *Bdnf*, which is known to be downregulated in RTT, showed a log2FC of −1.25 and an FDR of 1.05E-04 in cerebellum, while in blood, it was not detectable.

**Table 1:** Top 10 differentially expressed genes (DEGs) ordered by *p*-value in cerebellum and blood. In both tissues, we detected a strong downregulation of Mecp2, which acts as a positive control of the model and the methodology. This is also the case for Bdnf in cerebellum. Log2CPM represents the log2-average counts per million across all samples. Log2FC represents the log2-ratio Mecp2-null/wild type (WT); hence, positive values mean upregulation in Mecp2-null and vice versa.

Gene symbol	Log2CPM	Log2FC	FDR-corrected p-value
Cerebellum			
*Mecp2*	6.49	−3.88	5.38E-27
*Gm27640*	0.05	6.92	1.94E-23
*eLsm12*	4.5	1.91	5.39E-19
*Gpr21*	0.06	5.22	5.09E-16
*Gm10408*	−1.41	9.60	8.96E-13
*Zdhhc24*	3.96	1.31	1.17E-06
*Tenm2*	5.53	0.99	6.32E-06
*Bdnf*	4.2	−1.25	5.29E-05
*Paip2*	7.07	1.00	1.05E-04
*Gm3298*	−3.48	7.30	2.22E-04
Blood			
*Ube2v1*	5.71	2.58	4.52E-10
*Bpifa1*	4.78	15.53	2.70E-09
*Tmem164*	3.59	3.28	4.46E-09
*Tnnc2*	0.24	10.97	6.73E-09
*Mmrn1*	5.01	−3.25	4.73E-08
*Camp*	7.57	7.69	8.33E-08
*Scgb3a1*	3.88	14.63	1.75E-06
*Mpo*	4.54	10.18	1.75E-06
*Bace2*	−0.34	10.39	3.01E-06
*Mecp2*	3.85	−2.50	4.07E-06

Altogether, the comparison between blood and brain revealed high correlation of genes’ expression in both tissues.

### Identification of Overlapping Genes Between Cerebellum and Blood

**Figure 3:**
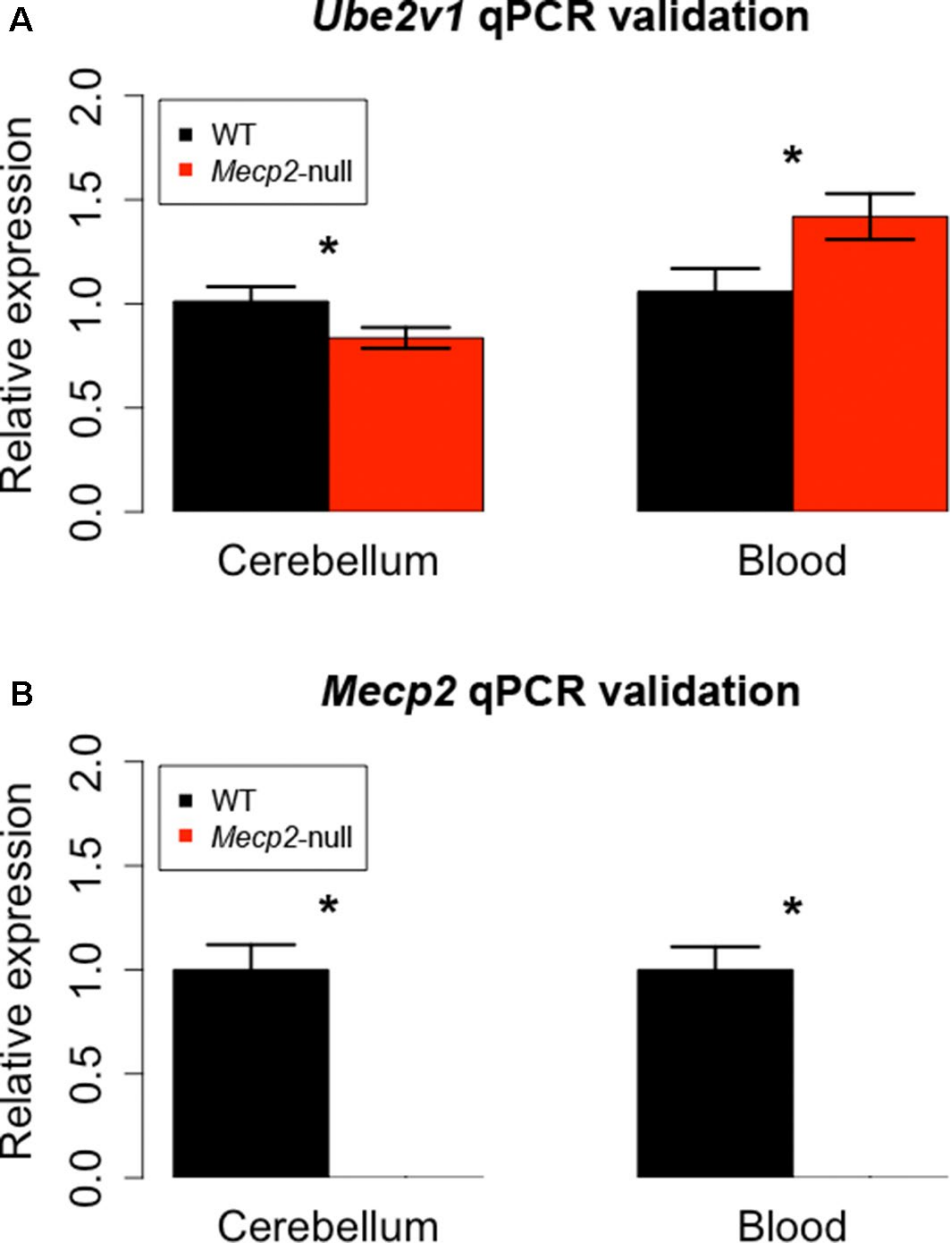
qPCR on different biological samples validate *Ube2v1* and *Mecp2* dysregulation in brain and blood. Validation by qPCR of *Ube2v1* **(A)** and *Mecp2* **(B)** differential expression between *Mecp2*-null (*N* = 6) and WT mice (*N* = 6). Expression is represented as relative expression, calculated as 2^ddCt^. Regarding *Ube2v1*, the ddCt between *Mecp2*-null and WT is 0.46 in blood (*p*-value = 0.012, FC = 1.42) and −0.27 in cerebellum ( *p*-value = 0.032, FC = 0.84). In *Mecp2*-null mice, no *Mecp2* expression was detected.

We then proceeded to identify DEGs present in both cerebellum and blood. We found two genes with an FDR-corrected *p*-value < 0.05 in both tissues: *Mecp2* and *Ube2v1*. *Mecp2* showed a log2FC of −3.88 and an FDR-corrected *p*-value of 5.38E-27 in cerebellum, and a log2FC of −2.50 and an FDR-corrected *p*-value of 4.07E-06 in blood. *Ube2v1* showed a log2FC of −2.91 and an FDR-corrected *p*-value of 0.02 in cerebellum, and a logFC of 2.58 and an FDR-corrected *p*-value of 4.52E-10 in blood. These genes were selected for quantitative polymerase chain reaction (qPCR) validation with additional samples (*n* = 12/group). As expected, *Mecp2* differential expression was confirmed, with no expression in the mutant samples and average delta Ct values (dCt) of 5.31 in cerebellum and 6.26 in blood. *Ube2v1* showed significant upregulation in blood of P50 *Mecp2*-null mice (ddCt = 0.46, FC = 1.42, *p*-value = 0.012), and it was downregulated in cerebellum of age-matched *Mecp2*-null mice (ddCt = −0.27, FC = 0.84, *p*-value = 0.032), (**Figure 3**). An analogous comparison in presymptomatic mice showed no significant difference between mutant mice and controls (cerebellum: ddCt = 0.058, *p*-value = 0.354, blood: ddCt = −0.354, *p*-value = 0.314). Additionally, RNA quantification performed in presymptomatic females did not reveal any differences of *Ube2v1* expression between heterozygous *Mecp2*-null and WT (data not shown). All this suggests that the differential expression of *Ube2v1* is linked to the appearance of the symptoms.

Our independent validation confirmed the dysregulation of *Ube2v1* in both brain and blood, identifying a particular form of ubiquitination as a mechanism broadly altered in *Mecp2* mutants.

### Gene Pathways and Network Analysis Reveals Mechanisms Dysregulated in Both Brain and Blood

To identify the cellular mechanisms differing between WT and mutant mice, we used the genes identified in the single gene analysis to perform pathway analysis, protein interaction network, and biological function analysis [gene ontology (GO)].

Potentially dysregulated pathways were identified with the iPathway software, which takes as an input differential expression data between two conditions and computes the overrepresentation and possible perturbation of biological pathways according to the differences in gene expression. The analysis revealed five significant (*p*-value < 0.05) pathways in cerebellum and 33 in blood. After FDR correction, the only significant pathway in cerebellum was complement and coagulation cascades and the only significant pathway in blood was platelet activation. The behavior of the complement and coagulation cascades pathway was driven by the dysregulation of the following genes: *Fga*, *Serpina1c*, and *Serpina1e*, while the affectation of the platelet activation pathway was driven by the genes: *Ptgs1*, *Mylk*, *P2rx1*, *Hpr2*, *Gp5*, *Vwf*, and *Actg1*. Interestingly, although there is no overlap between pathways in brain and blood, *Serpina1c* was identified by RNAseq to be DE both in brain and blood, so it was selected for validation with PCR (see paragraph below). The full list of genes with an uncorrected *p*-value < 0.05 can be found in **Supplementary File 2**.

The analysis of protein interaction network was performed with the STRINGapp in Cytoscape, which predicts interactions between the members of a protein input list, and related interactors. As input, we used the DE genes from cerebellum and blood separately. The software can identify which are the higher connected nodes in the network (“hubs”) and rank them according to the number of connections (degree).

**Figure 4:**
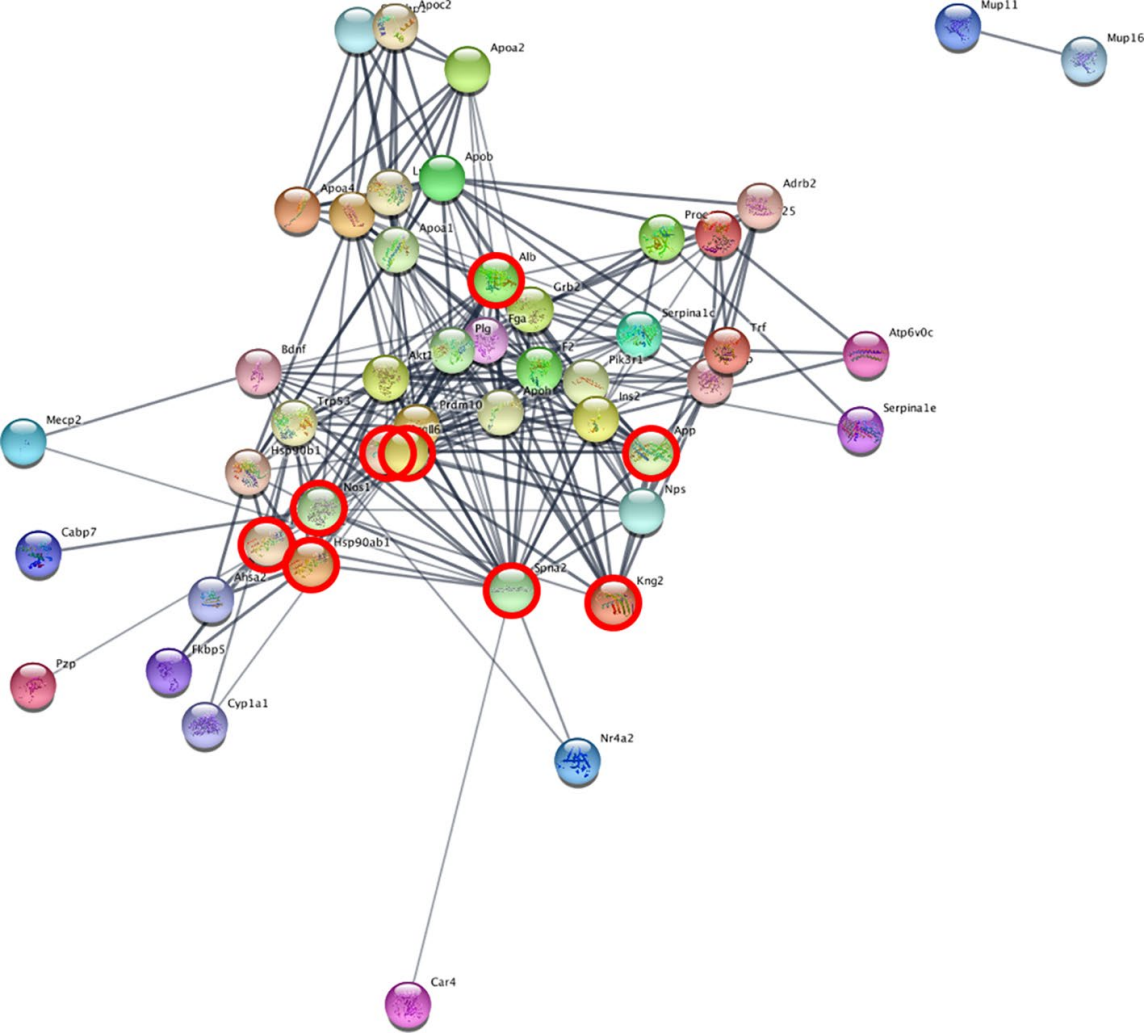
Network analysis reveals interacting genes across cerebellum and blood. STRING analysis network of cerebellum DE genes. The highlighted nodes correspond to proteins overlapping between blood and cerebellum.

We set up the analysis to add a maximum of 30 additional interactors per group. We then ranked the nodes by degree, in order to find proteins with the highest connectivity. In the cerebellum, the top scoring protein was Alb (Albumin), while in the blood it was Spna2 (Spectrin alpha 2). We then identified the network proteins present in both tissues, in order to find possible common mechanisms: Alb, App, Hsp90aa1, Hsp901b1, Ilt6, Kng1, Kng2, Nos1, and Spna2. **Figure 4** depicts the network obtained from cerebellum, with the proteins also present in the blood analysis highlighted. The full list of proteins and their interactors, ranked by degree, can be found in **Supplementary File 3**.

The common interactors are involved in the nitric oxide biosynthesis pathway, which in STRING results statistically significant (*p*-value = 0.016 after FDR).

**Figure 5:**
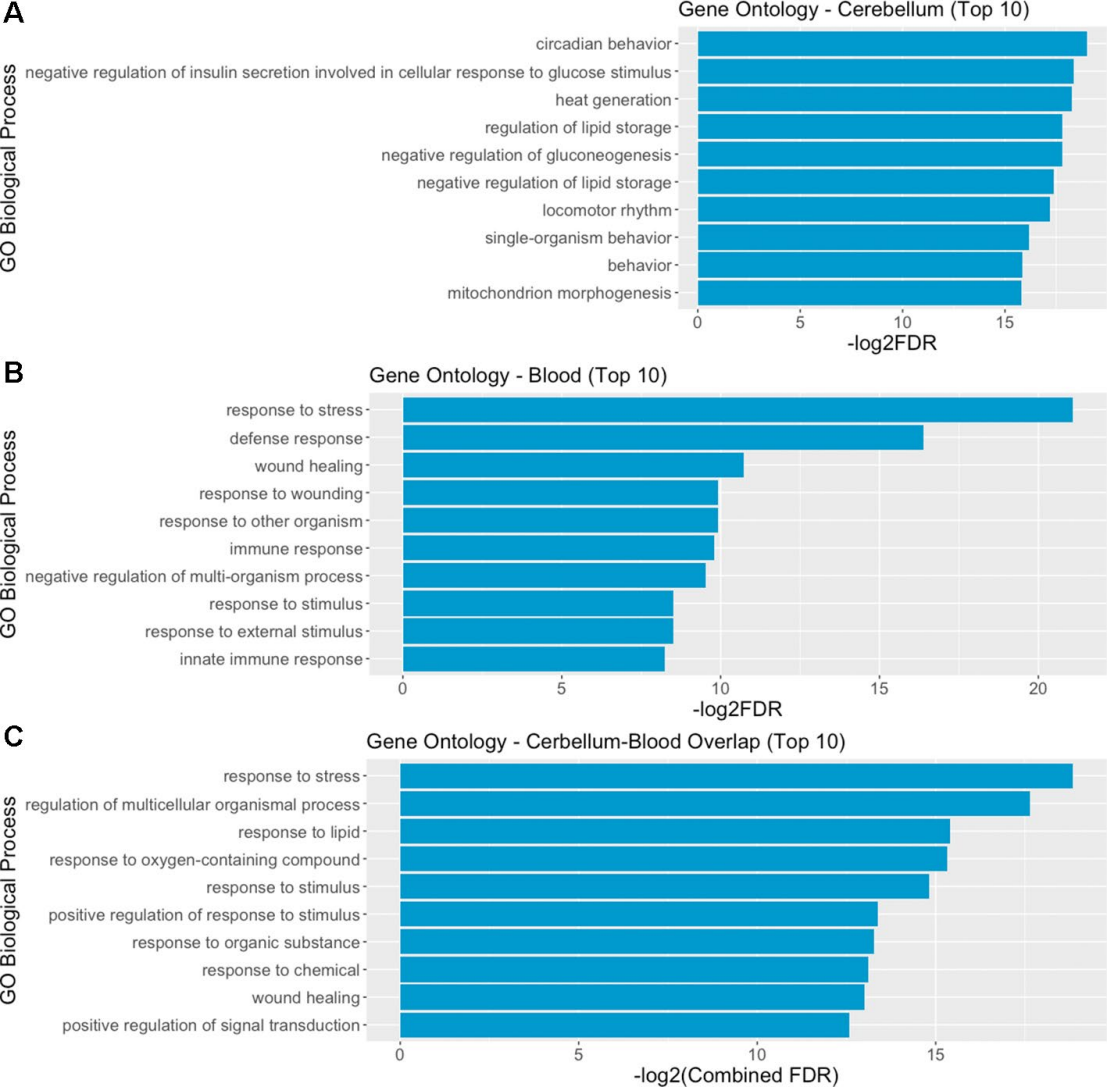
Gene ontology (GO) analysis reveals overlapping biological mechanisms in cerebellum and blood. **(A, B)** Top 10 gene ontology biological process categories enriched in cerebellum and blood DE genes and their interactors predicted by STRING. **(C)** Top 10 gene ontology biological processes overlapping between blood and brain, according to their combined FDR. Combined FDR was computed as follows: FDRCerebellum + FDRBlood − FDRCerebellum * FDRBlood.

We then performed a functional enrichment analysis on the output of the STRING analysis—including both DE genes and their interactors, in order to identify overrepresented gene ontology (GO) biological process categories. The analysis of functional enrichment revealed 462 and 477 significant GO categories in cerebellum and blood, respectively. The most significantly enriched process in the cerebellum is “circadian behavior,” while in the blood it is “response to stress.” The top 10 biological processes significantly enriched in cerebellum and blood are depicted in **Figure 5A and B**. We found 152 overlapping significant GO biological processes between cerebellum and blood, the most significant being “response to stress.” The top 10 most significant overlapping biological processes are depicted in **Figure 5C**. The full list of GO categories enriched in cerebellum, blood, and their overlap can be found in **Supplementary File 4**.

The gene-sets analysis reveals common mechanisms activated in blood and brain with an enrichment of mechanisms involved in system’s homeostasis, metabolic processes such as nitric oxide synthesis, and coagulation-related processes.

### qPCR Validation of Additional Genes

In addition to the identified overlapping genes in differential expression analysis, we considered for validation with qPCR additional genes that were selected according to different criteria. First, we selected genes that were significant in brain and also in blood and vice versa (**Table 2**). Of these, we tested with qPCR genes that were linked to the multiple gene analysis and/or were lined to mechanisms known to be associated to RTT: *Dnah14*, *Serpina1c*, *Lsm12*, *Mup10*, *Ankrd63*, *Hal*, *Ankrd63*, *Slc6a4*, *Crispld2*, *Tnnc2*, *Mpo*, *Gpx3*, *Paip2*, and *Fkbp5*. We also tested, in blood, genes that showed a high fold change or were dysregulated in cellular pathways in blood: *S100a9*, *Ms4a3*, *Snx31*, and *Prg3*, *Gstm2*, *Gsta3*, and *Itgb3*. Two of the selected genes (*Fkbp5* and *S100a9*) had already been identified by other screenings (18). The additional tested genes are reported in **Table 2**.

**Table 2:** Summary of additional genes tested by qPCR. The list includes differentially expressed (DE) genes overlapping between cerebellum and blood, some genes selected for their high fold change in blood and some genes selected from predicted dysregulated pathways. Log2FC refers to the ratio Mecp2-null/WT; hence, positive values mean upregulation in Mecp2-null and vice versa.

Additional genes tested by qPCR				
Genes identified in cerebellum that are also significant in blood				
Gene	log2FC cerebellum	FDR cerebellum	log2FC blood	Uncorrected *p*-value blood
*Dnah14*	2.04	2.51E-02	8.54	3.88E-03
*Fkbp5*	1.33	3.70E-02	1.40	4.90E-03
*Serpina1c*	−10.52	2.76E-02	9.87	5.50E-03
*Gm28374*	6.52	1.34E-02	−6.55	1.84E-02
*Lsm12*	1.91	5.39E-19	−1.30	3.24E-02
*Paip2*	1.00	1.06E-04	−1.06	3.49E-02
*Ahsa2*	−0.85	4.13E-02	−0.83	4.15E-02
*Mup10*	−10.75	3.30E-02	7.12	4.28E-02
Genes identified in blood that are also significant in cerebellum				
Gene	log2FC blood	FDR blood	log2FC cerebellum	Uncorrected *p*-value cerebellum
*Atp6v0d2*	10.74	4.30E-02	5.91	3.44E-04
*Hal*	9.73	2.87E-02	−5.88	3.65E-04
*Ankrd63*	8.90	3.79E-02	2.07	5.56E-04
*Hist1h2be*	−3.32	1.08E-02	−0.86	4.59E-03
*Slc6a4*	−1.99	2.27E-02	2.75	1.13E-02
*Crispld2*	2.54	4.87E-02	0.66	1.22E-02
*RP23-253I14.4*	11.74	1.05E-02	3.12	1.28E-02
*C430002N11Rik*	−8.55	5.89E-03	4.84	1.32E-02
*Tnnc2*	10.97	6.73E-09	3.44	1.37E-02
*Scgb3a2*	14.25	2.26E-03	4.57	1.91E-02
*Mpo*	10.18	1.75E-06	1.18	2.65E-02
*Gm5741*	−8.52	9.00E-03	2.67	3.33E-02
*Gpx3*	3.03	1.64E-02	0.73	3.37E-02
High fold change in blood				
Gene	log2FC RNAseq			FDR RNAseq
*S100a9*	2.87			2.01E-02
*Snx31*	−8.80			3.09E-03
*Ms4a3*	13.05			5.27E-03
*Prg3*	12.26			5.27E-03
Genes from predicted dysregulated pathways (blood)				
Gene	log2FC RNAseq			FDR RNAseq
*Gsta3*	8.98			4.06E-03
*Gstm2*	7.56			2.34E-04
*Itgb3*	−3.15			1.00E-03

We considered significantly different in the qPCR analysis those genes with a *p*-value < 0.05, and we considered “trending” the genes with a *p*-value between 0.1 and 0.05. The genes that confirmed to be dysregulated after qPCR are: *Serpina1c*, *Ankrd63*, *Crispld2*, *Mpo*, and *Gsta3*. The validation results are reported in **Supplementary File 5**xref>. In the case of *Serpina1c*, we performed an additional PCR in liver extracts as this gene is mostly expressed by hepatocytes (19). We confirmed the downregulation of *Serpina1c* in mutants also for this preparation (ddCt = −1.006, *p*-value = 0.008). We also tested the differential expression of *Serpina1c* between mutant and WT in cerebellum and blood of presymptomatic mice, obtaining negative results (cerebellum: ddCt = 1.804, *p*-value = 0.18, blood: ddCt = 3.563, *p*-value = 0.363). RNA quantification performed in presymptomatic females did not reveal any differences of *Serpina1c* expression between heterozygous *Mecp2*-null and WT (data not shown). Like in the case of *Ube2v1*, this suggests that the dysregulation of *Serpina1c* is linked to the appearance of symptoms.

Altogether, the multiple RNA measurements on different samples confirmed reveal that there are genes and mechanisms DE in both blood and brain.

## Discussion

The quest for understanding and treating neurodevelopmental disorders is hampered by several factors, one of these being access to the brain. Considering the limited molecular etiology, and the impact of *MECP2* mutations in multiple tissues to the clinical presentation (15), RTT represents an ideal model to study the mechanisms of disease present in both CNSs and peripheral systems, with the aim to identify markers in peripheral blood that would be accessible for diagnostic, prognostic, and treatment purposes.

In this study, we compare the expression of genes and molecular pathways in blood and brain of *Mecp2* mutant mice to identify common mechanisms dysregulated across different tissues. Since gene expression is strongly dependent on brain area and developmental stages, we first investigated which brain regions highly express the MeCP2 protein in P50 male mice—an age when symptoms are advanced. We find that in P50 mice, the cerebellum is the region with higher expression of MeCP2 compared to hippocampus, cortex, and hypothalamus (**Figure 1**), but protein expression in the cerebellum decreases in adult mice, confirming the results of Ross and colleagues (15). We used *Mecp2*-null male mutants and matched controls at P50, and we compared the DEGs in cerebellum to the DEGs in the blood. There are several studies that look at the gene expression profile in Rett patients (20) and mouse models (12, 14, 18, 21–39). However, to our knowledge this is the first study that compares directly differential gene expression in brain and blood of *Mecp2*-null mice versus matched controls.

The analysis of the expression levels shows a reasonable correlation between the genes expressed in the two preparations (R^2^_WT = 0.95, R^2^_MUT = 0.93, **Figure 2A**), although there is a higher variability in the blood compared to the cerebellum (**Supplementary Figure 6**). This variability could generate results less consistent in blood, reinforcing the necessity of validating the results with another method (PCR) on independent biological samples. Both the differential expression analysis (EdgeR) and the gene pathways and network analysis identify overlapping associations across the preparations.

**Figure 6:**
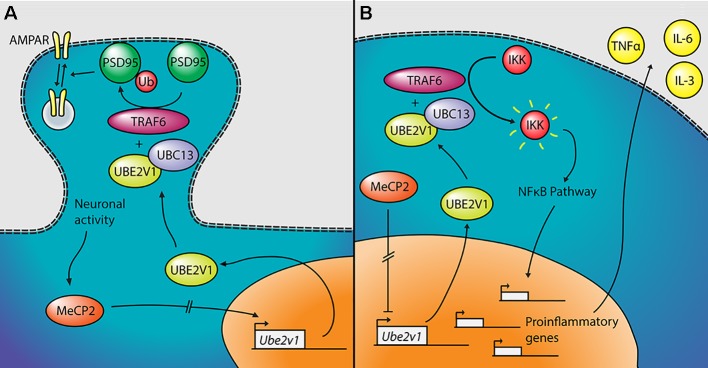
Proposed mechanism linking *Mecp2*, *Ube2v1*, and Rett syndrome (RTT). **(A)** In neurons, MeCP2 would upregulate the transcription of *Ube2v1*, directly or indirectly. UBE2V1, together with UBC13 and TRAF6, would promote Lys63-linked ubiquitination of PSD95, which would in turn regulate AMPA receptor (AMPAR) trafficking. **(B)** In peripheral blood mononuclear cells (PBMCs), MeCP2 would repress the transcription of *Ube2v1* (directly or not). UBE2V1, together with UBC13 and TRAF6, would activate IκB kinase (IKK), which would activate the NFκB pathway. This would promote the transcription of proinflammatory genes and the upregulation of cytokines such as TNFα, IL-6, and IL-3.

The most significant gene DE in the two tissues—other than *Mecp2* itself—is *Ube2v1* (also known as *Uev1a*), which is downregulated in cerebellum and upregulated in the blood of the mutant mice. Its differential expression was confirmed by qPCR both in the blood and in the cerebellum (**Figure 3**). *Ube2v1* encodes for Ubiquitin Conjugating Enzyme E2 V1, which is a ubiquitin-conjugating E2 enzyme variant (UEV) protein. UEVs are similar in sequence to ubiquitin-conjugating E2 enzymes but lack their enzymatic activity (40). This type of ubiquitination is not linked to proteolysis, but it acts as a system of nonproteolytic cell signaling instead (41) and has been associated to elements involved in synaptic plasticity and function. In the brain, the function of Ube2v1 is to modulate the protein organization at synaptic level and the ability of the neurons to respond to changes in activity (**Figure 5A**). **Figure 6** represents speculations regarding possible roles of Ube2v1 in RTT.

In a mouse study, postsynaptic density-95 (PSD95) and Ube2v1 were copurified using tandem affinity purification (42) and Ube2v1 mediates Lys63-linked polyubiquitination (L63-polyUb) of PSD95 in an activity-dependent and nonproteolytic manner. Such modification of PSD95 regulates two main properties associated to synaptic function: first, it affects PSD95’s scaffolding properties, promoting synaptic formation, maturation, and strength (43). Second, the ubiquitination of PSD95 is known to mediate N-methyl-D-aspartate-mediated α-amino-3-hydroxy-5-methyl-4-isoxazolepropionic acid receptor (AMPAR) endocytosis (44). Interestingly, the *Ube2v1* homologous *uev-1* regulates AMPAR trafficking in *Caenorhabditis elegans*, possibly by modulating a clathrin-independent AMPAR recycling pathway (45). Trafficking of AMPARs has been shown to be altered in the hippocampus of *Mecp2*-null mice (46). There is further evidence suggesting an important function of *Ube2v1* in the brain; knocking out its associate *Ubc13* in mouse results in impaired cerebellar synapse formation (47). This hypothesis, although only theoretical, suggests that *Mecp2*, through the activity-dependent regulation of *Ube2v1*, influences L63-polyUb of PSD95. PSD95 modulates synaptic maturation and function, which indeed are impaired in RTT (48).

Outside the brain, *Ube2v1* exerts control over cell cycle and differentiation (40), response to DNA damage through p53 (49), and regulates pathways responsible for immune inflammatory response, mainly through the nuclear factor kappa-light-chain-enhancer of activated B cells (NF-kB) pathway (50, 51) (**Figure 5B**). For instance, the UBE2V1–UBC13–TRAF6 complex activates nuclear factor of kappa light polypeptide gene enhancer in B-cells inhibitor (IkB) kinase (IKK) in response to proinflammatory cytokines (52, 53). This mechanism is consistent with the increased levels of NF-kB observed in MeCP2-deficient human peripheral blood mononuclear cells (PBMCs) and in the human monocyte line THP1 (54). The same authors found that the upregulation of NF-kB caused by MeCP2 deficiency enhances the expression of tumor necrosis factor alpha (TNFa), interleukin 6 (IL-6) and interleukin 3 (IL-3) (55), which can contribute to the subclinical immune dysregulation observed in RTT patients (56), including increased levels of TNFa and IL-6 in the blood, among other cytokines (57). Alteration of the NF-kB pathway was also suggested after transcriptomic analysis on the blood of RTT patients (58). NF-kB is also dysregulated in cortical neurons of *Mecp2*-null mice, with direct effects on dendritic complexity that can be rescued by reducing NF-kB signaling (59).

The presence of *Ube2v1* across different processes and cell types, and especially in the immune system, makes it an interesting candidate to examine the function of *MECP2* in the periphery and to reflect brain function in peripheral blood. A transcriptomic analysis performed in human PBMCs revealed enrichment of gene ontology categories related to regulation of protein ubiquitination (60). This could support the idea of *Ube2v1* also playing a role in the human condition.

### Serpina1c

*Serpina1c* resulted to be a DEG in both blood and brain. Its downregulation in the mutants was confirmed in cerebellum and showed a strong trend in blood. In blood, the qPCR expression analysis contradicts the one originally obtained by RNAseq, which indicated a strong upregulation in the blood of mutants. The results in the sequencing analysis though, are driven by one single sample, while the results in PCR have been replicated across 12 independent biological samples; hence, for this gene, we trust the decrease measured with PCR. The nature of *Serpina1c* gives reasons to pay attention to its possible involvement in RTT.

*Serpina1c* encodes for α-1-antitrypsin (α1AT) 1–3, which is part of the serine protease inhibitor (Serpin) family. While in mouse, *Serpina1* has five different variants (Serpina1a-e) with distinct specificity (61), in human, there is only one *SERPINA1* gene.

In mouse, the protein network for *Serpina1c* shows interactions with the elements of the Akt pathways, which has been shown to be dysregulated in *Mecp2* mutant mice (62).

In humans, mutations that result in a deficit of α1AT are mostly known to be a cause of pulmonary emphysema and liver disease (63). The liver is the main secretor of α1AT in both human and mouse, and it also expresses MeCP2. We confirmed the downregulation of *Serpina1c* RNA in the liver of mutant mice by qPCR. The MeCP2 and α1AT coexpression suggests a role of *Serpina1c/SERPINA1* in RTT.

In normal conditions, circulating α1AT protects the lungs by inhibiting neutrophil elastase, which degrades the connective tissue. Patients with low plasma levels of α1AT have an elevated risk of pulmonary emphysema, due to excessive degradation of the connective tissue. To our knowledge, respiratory deficiencies present in RTT have not been yet linked to this kind of pathology. However, emphysema-like features have been observed in the lungs of *Mecp2*-null mice (64).

The role of α1AT in liver pathogenesis would be less relevant in RTT, as its main disease-causing mechanism is the aggregation and accumulation of abnormal forms of α1AT in the hepatocytes (65, 66). The most intriguing result of the *Serpina1c* analysis is that diagnostic grade blood tests are already available to quantify the levels of circulating α1AT (67) and the same methods could be used in RTT patients for prognostic purposes.

Regarding its brain function, α1AT has been proposed to be involved in Alzheimer’s disease (AD) (68), Parkinson’s disease (PD) (69), schizophrenia (70), and amyotrophic lateral sclerosis (ALS) (71), but no specific mechanisms have been described. α1AT has been shown to drastically reduce excitotoxicity *in vitro* through the inhibition of calpain (72), but there is no evidence of this phenomenon being physiologically significant *in vivo*. α1AT is also therapeutic against stroke in rats (73).

It is also possible that Serpina1c in the brain acts through the interaction with other proteins. α1AT is an inhibitor of activated protein C (APC) (74). APC, in turn, neutralizes plasminogen activator inhibitor 1 (PAI) (75), which inhibits tissue plasminogen activator (tPA) (76). tPA is a protease that catalyzes the conversion of plasminogen to plasmin. Aside from its important anticoagulant role, plasmin is responsible for the cleavage of the inactive precursor brain derived neurotrophic factor (proBDNF) into active mature BDNF (mBDNF) (77). BDNF is considered relevant in RTT: it has been shown to be dysregulated in both RTT patients (78, 79) and in animal models (80, 81), and its overexpression in mice ameliorates the symptoms of *Mecp2*-null mice (80). MeCP2 seems to directly regulate the expression of BDNF in an activity-dependent manner (82), suggesting that the involvement of BDNF in RTT is transcription dependent. However, it is possible that the decreased expression of BDNF in RTT is dependent both by an MeCP2-dependent transcription, and by an abnormal posttranslational cleavage of BDNF. BDNF has been studied as possible peripheral biomarkers of mood disorders (83), and the tPA–BDNF pathway in serum is a target for the treatment of depression (84). Moreover, the fact that tPA’s main known role is related to hemostasis would be in accordance with the dysregulation of the platelet activation mechanisms, identified with the pathways analysis.

Interestingly, RNA expression analysis of both *Ube2v1* and *Serpina1c* in presymptomatic mice shows no difference between WT and mutant mice, suggesting that their altered expression may be linked to presentation of symptoms and severity of the condition.

#### Platelet Activation Pathway

Pathway analysis predicted a potential disruption of the platelet activation pathway in the blood of *Mecp2*-null mice. Interestingly, an altered coagulation pathway was identified in the brain of the same mutant mice, driven by the already discussed *Serpina1c*. If, according to the previously described hypothesis, the levels of plasmin were altered in RTT, an abnormal hemostatic state could be expected, although it has never been reported in patients.

Other mechanisms could potentially link platelet activation and RTT. In a metabolomic screening of *Mecp2*-null mice, an alteration of the platelet-activating factor (PAF) cycle was predicted (85). PAF is a multifunctional phospholipid, which acts through its G-coupled receptor PAFR (86) and is involved in activation of platelets and leucocytes and in synaptic function (87, 88).

Regarding its role in the synapse, PAF has been described as a retrograde messenger in hippocampal long-term potentiation (LTP) (89). PAF also mediates synaptic facilitation in striatal slices (90) and LTP in cortical slices (91). PAF can enhance presynaptic vesicle exocytosis through calcium signaling (92, 93). Animal models lacking PAFR have shown differing results, with a study claiming LTP attenuation (94) and another claiming a normal synaptic function (95). It has also been observed that PAF needs to be properly regulated: elevated levels of PAF can cause excitotoxicity (96), and it seems to be involved in various CNS diseases, such as AD, PD, epilepsy, stroke, or multiple sclerosis (97). LTP defects have been repeatedly observed in mouse models of RTT (46, 98–101), and PAF could be a contributing factor.

We speculate that the alteration of the platelet activation pathway could be influenced by abnormal levels of nitric oxide (described below), which has a limiting effect on platelet activation (102).

#### Nitric Oxide

Our protein interaction analysis revealed that some genes overlapping between blood and cerebellum could be related to the nitric oxide (NO) synthesis pathway. NO is a signaling molecule present in several biological processes. In the brain, it can act as an anterograde and retrograde neurotransmitter, and it can induce dendritic and presynaptic growth (103). As previously mentioned, synaptic function and morphology are abnormal in RTT. NO could play a role in anxiety (104)—which is characteristic of RTT (105)—and it has also been linked to other pathologies of the CNS such as schizophrenia, bipolar disorder, depression, autism, and fragile X syndrome (106, 107). Abnormal upregulation of neuronal NO synthase has been observed in enteric neurons of *Mecp2*-null mice (108). Conversely, a reduced NO availability has been observed to contribute to vascular dysfunction in *Mecp2*-null mice (109). Our data is not enough to describe if the NO levels are most likely to be up- or downregulated in our model, but it remains a hypothesis to explore further. NO would also be a potential target for a treatment. L-lysine, an inhibitor of NO synthesis, has been used in a trial as an adjuvant of risperidone in schizophrenia (110). Bumetanide, a molecule that has been shown to be effective in the treatment of ASD (111), has been suggested to achieve its effect by increasing the levels of NO (112), but no experiments have been performed in that regard.

The analysis of enriched biological functions with GO shows consistency with other function dysregulated in RTT. The enrichment of circadian behavior in the brain preparation is consistent with sleep disorders in patients with RTT (113), and with disruption found in *Mecp2* mutant mice. *Mecp2* mutants have altered nocturnal activity and present structural abnormalities of hypothalamic centers controlling circadian rhythms (114). In addition, both MeCP2 expression and the MeCP2-binding to promoters of regulated genes are correlated to circadian rhythms (115). The altered nocturnal activity correlates with anxiety behaviors and increased plasma concentration of corticosterone—the stress hormone—linking the enrichment of circadian behavior function to the increased response to stress function, present both in brain and in blood. Other functions that appear enriched in the GO analysis of blood and brain systems include metabolism, and response to stimuli such as immune response, which have been reported to be indeed altered in patients with RTT (116, 117).

Taken together, our findings point at several genes overlapping between brain and blood and connecting the multiple aspects of RTT. These results have implications not only for the understanding of the biological mechanisms of RTT and the broad action of MeCP2 across different tissues. In fact, the presence of peripheral markers associated to brain dysfunction and linked to the symptoms of RTT, would facilitate the monitoring of the disease and the evaluation of the functional effects of candidate treatments.

## Methods

### Mice

For screening the MeCP2 expression in control conditions, we used WT C57/BL6 male mice. For the sequencing experiment, we used *Mecp2* ^tm1.1Bird^ male mice available from Jackson (Stock no.: 003890) with a deletion of exons 3 and 4. WT C57/BL6 male mice were used as controls, as they match the background of the mutants. For qPCR, we used additional mice from the colony. Mice were genotyped using a standard PCR on DNA extracted from ear punches, using the protocol described in the Jackson website (www.jax.org). For the selection of presymptomatic and symptomatic mice, we used the criteria defined by Stearns et al. (118), where the authors run a battery to behavioral tests to define the onset of symptoms of RTT in male mice (after 28 days after birth—P28). For female mice, the onset of symptoms is after 3 months (P90). Mice were housed in the animal facility at 12 L/D cycle. All procedures on animals were authorized by the National Authority in Animal Welfare [Health Products Regulation Authority (HPRA)] Department of Comparative Medicine in Trinity College Dublin (TCD) (Authorization number: AE1936/I108, AE1936/P067).

### Protein Extraction and Enzyme-Linked Immunosorbent Assay (ELISA)

Tissue was harvested at postnatal ages of 4, 8, and 12 weeks. Cortex, hippocampus, hypothalamus, cerebellum, and full brain were dissected and stored at −80°C until protein extraction. Tissue was homogenized in radioimmunoprecipitation assay (RIPA) buffer (150 mM NaCl, 1% Triton X-100, 0.5% sodium deoxycholate, 0.1% SDS, 50 mM Tris-HCl pH = 8) containing cOmplete Protease Inhibitor Cocktail Tablets (Sigma), using pestles. The homogenate was incubated for 30 min in ice and then sonicated. The homogenate was centrifuged for 30 min at 20,000xG at 4°C, and the supernatant was stored at −80°C until further use. MeCP2 concentration was measured using a precoated sandwich ELISA assay (ELISAGenie). The MeCP2 concentration of each sample was normalized by its total protein concentration, measured using a Pierce^™^ BCA Protein Assay Kit (ThermoFisher). Statistical analysis was performed using a Kruskal–Wallis nonparametric test, and results were considered significant with a *p*-value < 0.05.

### RNA Extraction

Mice were sacrificed using CO_2_ at 8 weeks of age. Tissue harvesting was operated between 12 and 3 PM, and littermates were used as matching controls in the majority of cases (unless matched controls were not available). All the experiments comparing mutants and WT were run simultaneously. Cerebellums were dissected and stored at −80°C until RNA extraction. Blood was extracted immediately after euthanasia by suction from the heart and stored in RNAprotect Animal Blood Tubes (Qiagen). RNA was purified from brain and blood using the miRNeasy Mini Kit (Qiagen) and the RNeasy Protect Animal Blood Kit (Qiagen), respectively. RNA purity and concentration were assessed by the A260/230 and A260/280 obtained with a NanoDrop.

### RNAseq

Three *Mecp2*-null and 3 WT mice were used for RNAseq. RNA from blood was depleted from globin mRNA using a mouse/rat GLOBINclear^™^ kit (Ambion). RNA integrity and concentration were measured using a bioanalyzer. For the subsequent steps, only samples with an RIN (RNA Integrity Number) > 7 were used. Sequencing cDNA libraries were prepared using a NEBNext Ultra RNA Library Prep Kit for Illumina, along with a NEBNext Poly(A) mRNA Magnetic Isolation Module and the NEBNext Multiplex Oligos for Illumina (New England Biolabs). RNA (200 ng) were used as a starting material for each sample. Library concentration and mean fragment length were measured using a bioanalyzer. Libraries were pooled, and a preliminary sequencing run was performed in a MiSeq (Illumina). Then, they were sent for sequencing to Edinburgh Genomics, where a HiSeq 2000 system (Illumina) was used. Sixty million, 2x75bp paired-end reads were obtained for each sample. The quality of the reads was determined using FastQC. All reads had qualities above Q28 among their whole extent (a representative sample of the FastQC analysis is depicted in **Supplementary Figure 7**). Reads were aligned to the Ensembl GRCm38 mouse genome construct, using Hisat2 (119), with default parameters. Abundance tables were generated using Stringtie (119), using the –e and –B options (simplified protocol) and the rest of parameters on default. To obtain count tables, the output of Stringtie was processed with the prepDE.py script provided by the developers. To obtain fragments per kilobase of transcript per million mapped reads (FPKM) tables, the output of Stringtie was processed with the R package Ballgown. The gene expression tables were filtered by removing counts corresponding to miRNA and genes presenting 0 counts in all samples. Differential expression analysis was performed using EdgeR (120), a statistical package specifically designed to analyze transcriptomic data, and we selected a likelihood ratio test. Results were corrected for multiple testing. Genes were considered as DE with an FDR-corrected *p*-value < 0.05.

### Protein Interaction and Functional Enrichment Analysis

Protein interaction and functional enrichment analysis were performed in the STRINGapp plugin for Cytoscape (v.1.8.0), which uses information from the STRING database (string-db.org). DE genes obtained by RNAseq were used as an input. A maximum number of 30 maximum additional interactors was selected. The confidence score cutoff was set at 0.6. Functional enrichment analysis was also performed on Cytoscape, using the STRING Enrichment plugin.

### Pathway Analysis

Pathway analysis of the results obtained by RNAseq was performed by using the iPathway software (Advaita). The software generates the *p*-value associated to each result considering the correction for multiple testing.

### Identification of Common Differentially Expressed Genes in Blood and Cerebellum

Identification of the significant DEG in each tissue was performed selecting the significant genes identified by EdgeR after multiple testing correction (*P* value ≤ 0.05). For the selection of DEG in both blood and brain, we used the list of DEG in one tissue (i.e., brain) and we tested the hypothesis that they were also significant in blood with the appropriate correction. We repeated the procedure for the DEG in blood also significant in brain.

### qPCR

Six *Mecp2*-null and 6 WT mice were used for qPCR. RNA was reverse transcribed using the Quantinova RT Kit (Qiagen). Reactions (20 µl) were set up, using 2x Gene Expression Master Mix (Applied Biosciences) and PrimeTime^®^ qPCR Probe Assays (Integrated DNA Technologies). We used the following primer–probe sets:

- *S100a9*: F: GGAATTCAGACAAATGGTGGAAG R: CATCAGCATCATACACTCCTCA; probe:/56-FAM/TGACATCAT/ZEN/GGAGGACCTGGACACA/3IABkFQ/

- *Ms4a3*: F: TCAATACCCAGGCTTTCAAGG R: GAGAATCAGCATTAAAGACACCAG; probe:/56-FAM/TGCAGACAT/ZEN/CAGGTGACGGTGAAG/3IABkFQ/

- *Serpina1c*: F: GGAATCACAGAGGAGAATGCT R: GAATAAGGAACGGCTAGTAAGACT; probe:/56-FAM/TGTGCATAA/ZEN/GGCTGTGCTGACCA/3IABkFQ/

- *Lsm12*: F: CCTAGCTTCACTCAATGTTAGTAAG R: ATGGTCTTGTGAATGGTCTGG; probe:/56-FAM/TCAGCTTCT/ZEN/CCTCCTTCTCCGTCC/3IABkFQ/

- *Ankrd63*: F: CCAGCTTGATTTCCTTGTCCT R: CCTGAGCCATCCACCTTTC; probe:/56-FAM/AGAAGCAGC/ZEN/CGTTGTTCACACCT/3IABkFQ/

- *Crispdl2*: F: TCTGAGTGTCCATCCAGCTA R: TTCCACCTCGTTCATCATATCC; probe:/56-FAM/AGAAGCAGC/ZEN/CGTTGTTCACACCT/3IABkFQ/

- *Scgb3a2*: F: CTGGTATCTATCTTTCTGCTGGTG R: GTCGTCCAAAGGTACAGGTAA; probe:/56-FAM/TGGTTATTC/ZEN/TGCCACTGCCCTTCT/3IABkFQ/

- *Prg3*: F: CTATGTGCTGGTGAGGACTC R: AACTATAACTGTGGACGGAAGC; probe:/56-FAM/ATCTCCTGC/ZEN/AGACTCTCTGAGCCT/3IABkFQ/

- *Fam69c*: F: TGAGCCATTTCGACAGTGAC R: CCATGTCTACGTCAATAGCTACC; probe:/56-FAM/TGATGTCAA/ZEN/ACCTGAGAACTTCGCCA/3IABkFQ/

- *Has2*: F: AGTCATGTACACAGCCTTCAG R: GACCTTCACCATCTCCACAG; probe:/56-FAM/CATAATCCA/ZEN/CGCTTCGCCCCAGT/3IABkFQ/

- *Vmn2r85*: F: CCACAGAGTCAACAACTTCA R: GTACATGTCACACTGCACATTG; probe:/56-FAM/ATGGGCCAC/ZEN/AGGAGGAACATCAG/3IABkFQ/

*- Acc2os*: F: CATCCCTCCTGTTGTTATTATTCATC R: TCTGCTCCACTGAGTTTACTG; probe:/56-FAM/AGCTAAGCC/ZEN/TGGTTCCTTTGTTCCTG/3IABkFQ/

*- Gapdh*: F: AATGGTGAAGGTCGGTGTG R: GTGGAGTCATACTGGAACATGTAG; probe:/5Cy5/TGCAAATGGCAGCCCTGGTG/3IAbRQSp/

*- Slc14a2*: F: CAACCGCATCTACTTCCTGAC R: GCTCTCTTCTGCCTTCCAC; probe:/56-FAM/ACTGCTCTC/ZEN/CACTGCCACCATT/3IABkFQ/

*- Snx31*: F: CCAGATGAGCAGAGTGAAGTG R: CTAGGTTCTGGTTGAGAGTTCG; probe:/56-FAM/AGCAGAGTT/ZEN/CCAAGGAAAGTGACCTG/3IABkFQ/

- *Bpifa1*: F: CCTCTCCTGAACAACATCCTC R: AGACTTCCAACTACGGGCATA; probe:/56-FAM/CCATCGTCT/ZEN/CTATGTCACCATCCCTCT/3IABkFQ/

- *Tfap2d*: F: TGAGCCAGGATAGATCACCA R: GCTTAGAGCTGCACATATTGC; probe:/56-FAM/CCAGACCCA/ZEN/CTCCCATTCTAGACCT/3IABkFQ/

- *Ube2v1*: F: CACTTACAAGATGGACAGGCA R: GGTACTTAGGCCCACACTCTA; probe:/56-FAM/ACCTCCACG/ZEN/AACAATCTATGAAAACCGAA/3IABkFQ/

- *Dnah14*: F: TCAGTATAGAAGTCCTCTCAGTCA R: TGCACACGACATATTGATCCG; probe:/56-FAM/CCAGTACGA/ZEN/ACCTGACAGAATAGCTGC/3IABkFQ/

- *Mup1*: F: TGAGAAGCATGGAATCCTTAGAG R: ATGAACACCAACCCACTCC; probe:/56-FAM/TATCCAATG/ZEN/CCAATCGCTGCCTCC/3IABkFQ/

- *Atp6v0d2*: F: AGTCTTACCTTGAGGCATTCTAC R: GCCAAATGAGTTCAGAGTGATG; probe:/56-FAM/TCCCATTCT/ZEN/TGAGTTTGAGGCCGAC/3IABkFQ/

- *Hal*: F: CCATCAGAAATCGCAGAAAGC R: AGTTCTGTAGTGATGATGTCCTTC; probe:/56-FAM/CGTACACCT/ZEN/TACGCTGCTGTCCAC/3IABkFQ/

- *Hist1h2be*: F: CGCAAACGCTACTGAAAGGA R: TTCTTGCCGTCCTTCTTCTG; probe:/56-FAM/TCTGAAGAT/ZEN/GCCTGAGCCAGCC/3IABkFQ/

- *Slc6a4*: F: CATCGTCTGTCATCTGCATCC R: CGTTGGTGTTTCAGGAGTGAT; probe:/56-FAM/TCCTTAAGT/ZEN/GTCCCTGGAGTGCTGA/3IABkFQ/

- *Tnnc2*: F: GAGTGCGGAGGAGACAAC R: CCATCAGCATCGAACATGTCA; probe:/56-FAM/AACCATGAC/ZEN/GGACCAACAGGCT/3IABkFQ/

- *Mpo*: F: CCCGCATTCCTTGTTTTCTG R: GCTTCTCCCCATTCCATCG; probe:/56-FAM/CTCACCTCC/ZEN/ATGCACACCCTCTTT/3IABkFQ/

- *Gpx3*: F: GCAGTATGCAGGCAAATATATCC R: CCCAGAATGACCAAGCCAA; probe:/56-FAM/TCTGTCAGA/ZEN/CCTCAGTAGCTGGCT/3IABkFQ/

- *Paip2*: F: GACAGGATTCGTTGGCTACC R: GACTTGGATCTTTCATGGTTGG; probe:/56-FAM/TCGTTGTCG/ZEN/TTTTTAACCCAGTGCAC/3IABkFQ/

qPCR was performed in a Quantstudio 5 Real Time PCR System (Applied Biosciences), using the following cycle: 2 min at 50°C, 10 min at 90°C, and 40x (15 s at 95°C and 1 min at 60°C). All samples were analyzed in triplicate, and *Gapdh* was used as a loading control. For target genes, we used the reporter fluorophore fluorescein amidite (FAM), and for *Gapdh*, we used Cy5. For each sample, the average *Gapdh* Ct value was subtracted from the average target Ct value, obtaining a dCt value. The average dCt of the WT group was used to calculate the ddCt value for each sample. Statistical significance was assessed using a *t*-test on the ddCt values, in Microsoft Excel. Significance was considered with a *p*-value < 0.05.

## Data Availability Statement

The datasets generated for this study can be found in the Gene Expression Omnibus, under accession number [GSE129387].

## Ethics Statement

This study was carried out in accordance with the recommendations of National Animal Welfare Authority, Ireland. The protocol was approved by the Animal Ethical Committee Trinity College Dublin and HPRA.

## Author Contributions

AS performed the experiments and wrote the paper; KH provided assistance in the design and analysis of the RNAseq experiment; DT contributed to sample extraction and establishment of the colony; and DT and MG designed and supervised all the parts of the research and the writing of the manuscript.

## Funding

The study was funded by the Wellcome Trust Grant WT079408/C/06/Z issued to MG, and by an SFI FN Funded Investigator grant 208377, and an IRSF grant 207417 issued to DT.

## Conflict of Interest Statement

The authors declare that the research was conducted in the absence of any commercial or financial relationships that could be construed as a potential conflict of interest.
