# Supplementary material for: Transcriptomic Analysis of Mecp2 Mutant Mice Reveals Differentially Expressed Genes and Altered Mechanisms in Both Blood and Brain
Source: Front Psychiatry. 2019 Apr 29;10:278. doi: 10.3389/fpsyt.2019.00278 (PMC6501143; doi:10.3389/fpsyt.2019.00278)
Supplement: Supplementary file 6 [file Image_1.pdf]

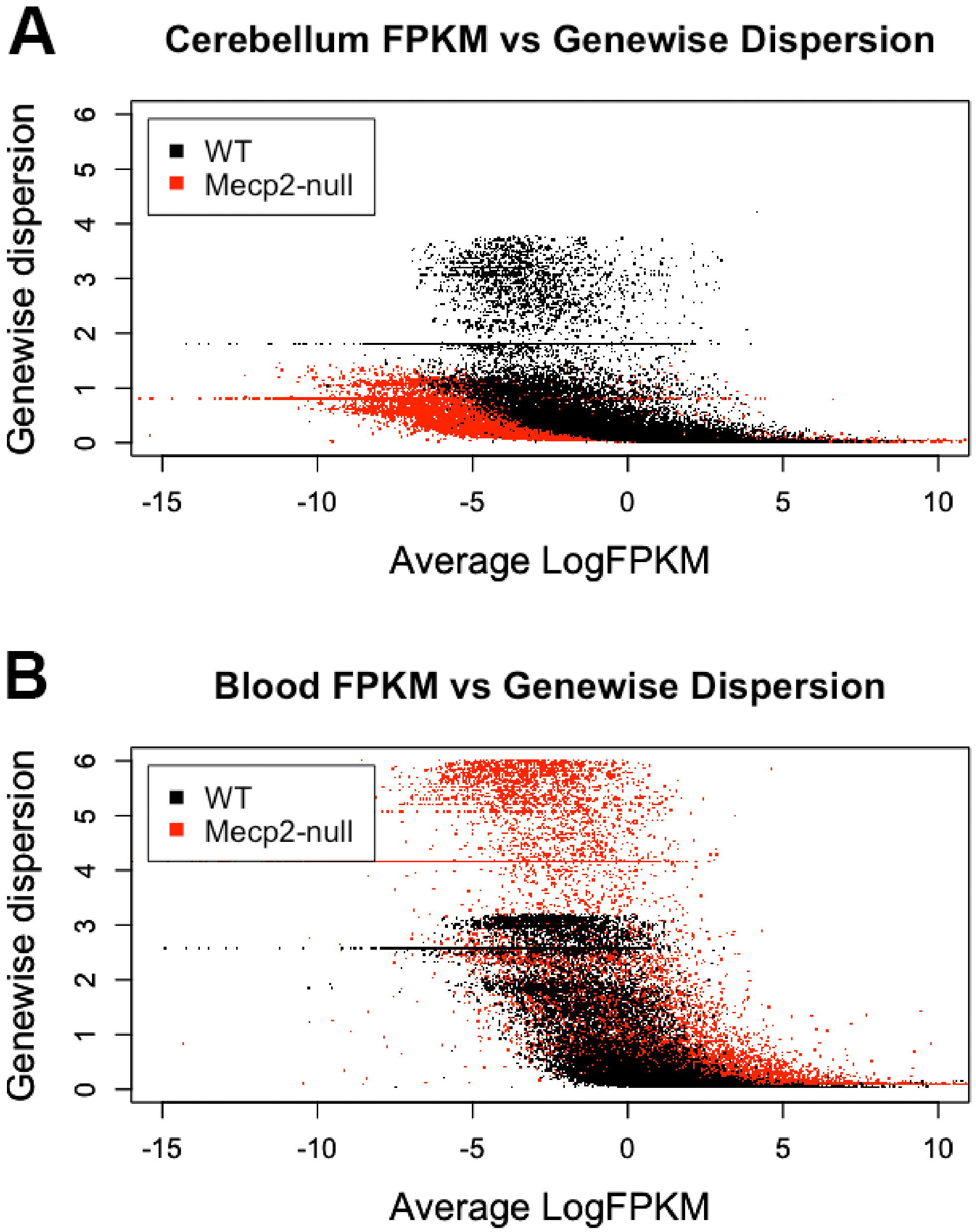

Supplementary Figure 1. Scatterplot of average gene expression (in logFPKM) versus genewise dispersion in cerebellum (A) and blood (B). Black and red colours represent WT and Mecp2-null data, respectively.
