# Supplementary material for: Transcriptomic Analysis of Mecp2 Mutant Mice Reveals Differentially Expressed Genes and Altered Mechanisms in Both Blood and Brain
Source: Front Psychiatry. 2019 Apr 29;10:278. doi: 10.3389/fpsyt.2019.00278 (PMC6501143; doi:10.3389/fpsyt.2019.00278)
Supplement: Supplementary file 7 [file Image_2.pdf]

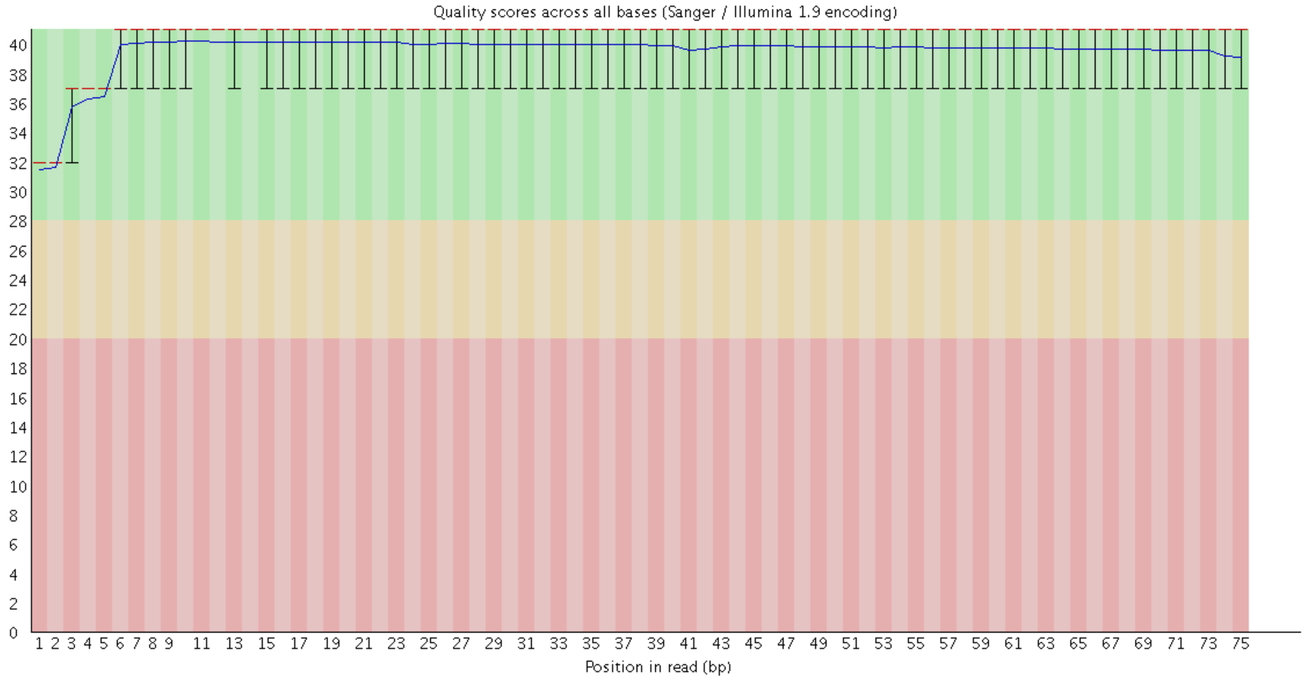

Supplementary Figure 2. Representative example of quality control of the sequencing reads obtained using FastQC. The y axis represents the mean quality score for each read position. Values in the green area are considered of good quality.
